# Complete Genome Sequence of a Reference Stock of Simian Immunodeficiency Virus RNA (SIVmac251/32H/L28) Determined by Deep Sequencing

**DOI:** 10.1128/genomeA.00344-16

**Published:** 2016-05-26

**Authors:** Adrian Jenkins, Claire Ham, Neil Almond, Neil Berry

**Affiliations:** Division of Virology, National Institute for Biological Standards and Control, South Mimms, United Kingdom

## Abstract

A reference preparation for simian immunodeficiency virus (SIV) RNA nucleic acid assays was characterized by complete genome deep sequencing. The entire coding sequence and flanking long terminal repeats, including minority species, were determined. This information will inform SIV research investigations and aid evaluation and development of amplification assays for SIV RNA quantification.

## GENOME ANNOUNCEMENT

Model systems to evaluate putative vaccine strategies and pathogenesis studies of HIV/AIDS employ strains of simian immunodeficiency virus (SIV) in experimental design. In cynomolgus macaques (*Macaca fascicularis*), a high-titer bulk SIV positive plasma stock recovered at the peak of virus replication *in vivo* was developed into an SIV RNA reference panel (C1−C5), based on the SIVmac251/32H/L28 isolate (1, 2). These represent commutable materials for determination of SIV viral load (2). The genetic composition of the highest viral concentration member of this panel (C1) was determined by deep sequencing analysis. The genetic composition of this SIV RNA reference material has not been described; here, we report the previously undescribed complete genome sequence using deep sequencing approaches, which are analogous to those previously reported for HIV-1 and HIV-2 (3, 4).

The C1 preparation represents a 1/100 dilution of undiluted plasma, from which viral RNA was extracted using QiaAMP viral RNA minikits (Qiagen). Reverse transcription and amplification reactions were performed with the SuperScript III One-Step RT-PCR system with Platinum Taq HiFi (Invitrogen) using primer pairs designed to generate five overlapping amplicons. Primers were designated as follows: 1F: CAGTCGCTCTGCGGAGAGGCTGGC; 1R: CAAGCTTAGTACTACTGGTCTCCTCCAAAGAG; 2F: TAGCTGTCTTTTATCCAGGAAG; 2R: TCTAATTAACCTACAGAGATGTTTGG; 3F: AAAATTGAAGCAGTGGCCATTAT; 3R: TACTTATGAGCTCTCGGGAACCT; 4F: GGCATAGCCTCATAAAATATCTG; 4R: GCGAGAAAACCCAAGAACCCTAG; 5F: CAGTCACCATTATGTCTGGATTG; 5R: CAAGAAAGTGGGCGTTCCCGA.

Bar-coded libraries of amplicons were made using Nextera XT library preparation kits (Illumina), and equimolar amounts of each sample were sequenced in 250-bp paired-end MiSeq sequencing reactions. After removal of primer and adaptor sequences, *de novo* assembly was performed on quality-trimmed and paired reads using Geneious version 7 software. Resultant contiguous sequences were aligned with the SIVmm32H reference sequence (D01065), and a consensus sequence was generated. All reads were mapped against the consensus to establish read depth and minority species.

The sequence of the SIV RNA reference material described here is 10,276 bp long with a G+C content of 44.0%, representing the complete coding sequence with nine open reading frames (*gag*, *pol*, *vif*, *vpr*, *tat*, *rev*, *vpx*, *env*, and *nef*), as well as the U5 region of the 5′-long terminal repeat (5′-LTR) and U3 and R regions of the 3′-LTR of the SIV genome. BLAST analysis (5) of the consensus RNA template-derived sequence further confirmed the highest similarity with the SIVmm32H proviral clone sequence (4) (total score 18,384, 98% identity, and 100% coverage). The mean read depth was 74,051.7-fold (±87,072.4 standard deviation) with a minimum of 16,285-fold. There are 39 positions with a minority nucleotide differing from the reference sequence with a frequency >3% and a base call accuracy of 99.9% (>Phred 30). In addition, there is one insertion event and one deletion event.

This is the first report of the complete genome sequence of the SIV RNA reference panel (CFAR catalogue no. 100092) derived from a viral RNA template, which will augment the development and evaluation of SIV RNA amplification assays applied in SIV vaccine and pathogenesis studies.

### Nucleotide sequence accession number.

The complete genome sequence of the C1 SIVmac251/32H/L28 reference used in the reference panel reported here has been deposited in GenBank under the accession number KU892415.
